# Yeast Protein as an Easily Accessible Food Source

**DOI:** 10.3390/metabo12010063

**Published:** 2022-01-11

**Authors:** Monika Elżbieta Jach, Anna Serefko, Maria Ziaja, Marek Kieliszek

**Affiliations:** 1 Department of Molecular Biology, The John Paul II Catholic University of Lublin, Konstantynów Street 1I, 20-708 Lublin, Poland; 2 Department of Applied Pharmacy, Medical University of Lublin, Chodźki Street 4a, 20-093 Lublin, Poland; annaserefko@umlub.pl; 3 Institute of Physical Culture Studies, Medical College, University of Rzeszów, Cicha Street 2a, 35-326 Rzeszów, Poland; mziaja@ur.edu.pl; 4 Department of Food Biotechnology and Microbiology, Institute of Food Sciences, Warsaw University of Life Sciences-SGGW, Nowoursynowska Street 159C, 02-776 Warsaw, Poland

**Keywords:** yeast protein, single cell protein, nutritional biomass

## Abstract

In recent years, the awareness and willingness of consumers to consume healthy food has grown significantly. In order to meet these needs, scientists are looking for innovative methods of food production, which is a source of easily digestible protein with a balanced amino acid composition. Yeast protein biomass (single cell protein, SCP) is a bioavailable product which is obtained when primarily using as a culture medium inexpensive various waste substrates including agricultural and industrial wastes. With the growing population, yeast protein seems to be an attractive alternative to traditional protein sources such as plants and meat. Moreover, yeast protein biomass also contains trace minerals and vitamins including B-group. Thus, using yeast in the production of protein provides both valuable nutrients and enhances purification of wastes. In conclusion, nutritional yeast protein biomass may be the best option for human and animal nutrition with a low environmental footprint. The rapidly evolving SCP production technology and discoveries from the world of biotechnology can make a huge difference in the future for the key improvement of hunger problems and the possibility of improving world food security. On the market of growing demand for cheap and environmentally clean SCP protein with practically unlimited scale of production, it may soon become one of the ingredients of our food. The review article presents the possibilities of protein production by yeast groups with the use of various substrates as well as the safety of yeast protein used as food.

## 1. Introduction

With the global human population explosion, the demand for food increases rapidly, especially for protein products. The world’s population is estimated to increase by 2 billion people in the next 30 years, from 7.7 billion currently to reach 9.7 billion by 2050 and could peak at nearly 11 billion around 2100, of which about two-thirds are supposed to live within urban areas [1]. Population growth in combination with rapidly increasing demand for meat nutrition are creating a protein deficit between the meat available and the expected demand in 2050 and following years. Global demand for meat as a protein source is steadily increasing. Over the past 50 years, meat production was increased more than three times. Each year, 80 billion animals are slaughtered for meat. Throughout the world, the average person consumes nearly 43 kg of meat a year. However, richer people eat much more meat, about 80 kg in Europe and even over 100 kg in the US and Australia [2]. Additionally, numerous studies showed that there is the association between consumption of red meat or processed meat and cancer risk. Large consumption of red meat significantly increases the risk of colon and rectum cancer, and some studies also demonstrated that red meat consumption is associated with some other cancers, such as pancreatic and prostate cancer [3]. Moreover, high levels of animal protein intake may also significantly increase the risk of premature mortality from cardiovascular diseases, and type 2 diabetes [4].

**Figure 1:**
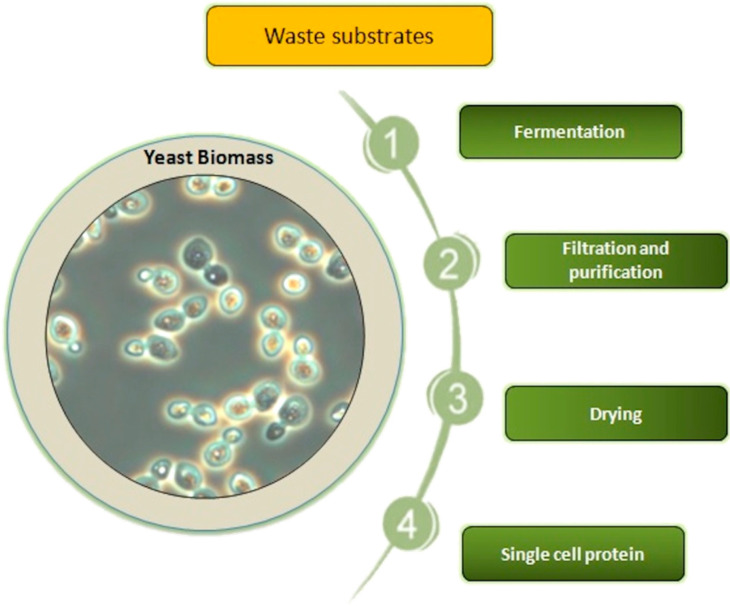
Production of single cell protein by yeast.

In about half of African countries, people consume as low as 10 kg meat per person a year [2]. In these countries, there is protein source deficiency which is beginning to be a public health problem [5]. However, shortage of protein sources such as meat, dairy, and plant protein would be limited not only to developing countries in Africa, Asia, and South America, but can also become a problem in more advanced countries in the future [6]. Therefore, one of the world’s most urgent challenges for the growing population is the production of easily accessible protein products in such a way that they do not have a negative influence on life. Furthermore, people from advanced countries are interested in developing healthier food, with optimal composition of amino acids and good quantity and quality fat, produced in an environmentally friendly manner [7]. Plants are nutritionally valuable protein sources (they may represent more than 50% of the protein used in animal feed), but require many acres of arable land and lots of water [8]. Moreover, currently, soybean meal is a main protein source for feedstock production, but it is suggested to replace plant feed with microbial biomass including post-fermentative yeast biomass in animal diets up to 100% [9]. In the case of animal protein, the time to obtain it is much longer, and it does not have a differentiated amino acid profile compared to yeast protein. Animal proteins are not available at reasonable prices to all people around the world [8]. Therefore, the need for protein leads to scientific challenges aimed at designing new nutritional strategies and the search for alternative nutritional sources of protein. Obtaining single cell proteins from yeast cells obtained from cultivation in waste products from various branches of the agri-food industry may be particularly important in the case of traditional agriculture [10]. It is very important to conduct scientific research aimed at obtaining the highest efficiency of protein production and an appropriate amino acid profile in yeast cell biomass. Thus, obtaining a balanced, renewable high-protein ingredient is an interesting alternative to classic protein products. Therefore, the answer to humankind’s challenge to meet the need of protein products seems to be the protein produced by various microorganisms such as bacteria, yeast, algae, and fungi. This microbial protein is also called bioprotein, protein biomass, or single cell protein (SCP), though filamentous algae and fungi may be multicellular [7,11]. SCP is dead and dried biomass of microorganisms which culture on various carbon and energy sources (Figure 1). SCP agricultural, food, and industrial residues and wastes are potential substrates for microbial protein production [12,13,14]. Obtaining microbial protein by conversion of waste substrates to value-added feed and food as high nutritional protein biomass and thus reduction of environmental pollutions is very important valuable feature of SCP production [14]. Particularly, yeast plays a special role in purifying the environment from waste materials, especially oleaginous yeast such as *Yarrowia lipolytica* and *Candida* spp. that are capable of growing on many industrial wastes including alkanes, petroleum by-products, natural gas, glycerol, biofuel waste, and plant or animal-waste fats [11,12,15,16,17,18,19]. The oily waste biodegradation by this yeast has a significant importance for environmental protection [16,18,20,21,22]. Production of fuels such as petrol and biodiesel generate huge amounts of residues, by-products, and wastes which cannot be utilized in these production processes [23]. One of the methods of getting rid of oily waste products after processing petroleum is its use as a culture medium for the production of added value compounds, such as protein, by yeast that can utilize these wastes [18,24,25]. Furthermore, both food grade or industrial wastes as well as forestry and agricultural sources are easily available and low- or even free-cost substrates as carbon and energy sources for SCP production by yeast [7,11,17,18,26,27,28,29].

On the market of some countries, currently, there are: some food spreads made of yeast extract, especially bakery or brewery *Saccharomyces cerevisiae*; food supplements containing brewery or bakery yeast or algae; a meat substitute product provided from filamentous fungus *Fusarium venenatum* and yeast *Torula* (*Candida utilis*, renamed as *Pichia jadinii*); *Pichia* and *Kluyveromyces* used as flavoring agent [7,30]. However, microbial protein biomass has little contribution to human and animal nutrition; hence, the increasing global protein demand is probably going to make SCP growing very important for the increasing world population

The following review provides information about yeast protein production using different examples of substrates (natural wood wastes, agricultural wastes, biofuel waste), and yeast species used as protein biomass producers and nutritional benefits. The review also presents a series of activities aimed at the use of SCP in food technology.

## 2. Production of Yeast SCP from Specific Waste Substrates

Yeasts, being living microorganisms, are not very demanding in terms of living conditions; therefore, they are considered to be widespread in the natural environment. The number of discovered yeasts increases every year; so far, only 1500 species have been known. This is 1% of their entire population. It is estimated that there may be up to 150,000 organisms belonging to the kingdom of fungi (Fungi) [31]. Some of them, due to their properties, play an important role in industrial sectors. Yeast is unicellular eucaryotic microorganism classified in the kingdom fungi of the phylum *Ascomycetes*; class: *Saccharomycetes*, which is also named *Hemiascomycetes*. The conventional true yeast *S. cerevisiae* is separated into one order *Saccharomycetales*. Yeast multiplies as single cells that divide by budding, e.g., *Saccharomyces*, or direct division by fission, e.g., *Schizosaccharomyces* [32].

SCP is obtained in a shorter period of time, much easier and cheaper to cultivate and harvest than crops or livestock. Moreover, yeast is flexible in the utilization of a variety of substrates. As shown in Table 1, common sources of yeast growth substrates for protein biomass production are represented by wide range of hydrophilic and hydrophobic wastes and by-products, biomass, and raw materials. According to several authors [33,34], fatty substrates favor a high-protein concentration in yeast biomass.

The cellulose, hemicellulose, and lignin as natural wood wastes are inexpensive or cost-free and easily accessible sources for protein production, but they must be pretreated enzymatically [69,72]. Domestic sewage and wastes originating from food processing, as well as wastes and by-products from starch production and other agricultural wastes can also be utilized easily [72]. It is best to choose such waste substrates that are cheap or cost-free and also easily accessible. Using these substrates by the yeast protein producers contributes to the reduction of pollution. Sometimes, it is required to add other nutrients such as nitrogen, phosphorus, and others into a waste culture medium to support optimal growth of the selected strains [73]. It is observed that agricultural wastes are very good substrates for cost-effective protein production, resulting in a good quantity and quality of yeast protein biomass which is delicious for livestock [72,74]. Yeast protein biomass obtained from various industrial wastes, including petroleum by-products and biofuel waste are also suitable for animal feeding [72,74,75]. However, yeast protein biomass can only be consumed by humans after further food processing in line with local flavors [72].

During the protein synthesis, nitrogen is one of the significant factors due to protein structure properties. Nitrogen sources useful for microbial growth include ammonia, ammonium salts, urea, and organic nitrogen in a variety of media as exemplified by industrial waste materials [74,76]. The best nitrogen source is tryptone and yeast extract. When tryptone was supplemented at a concentration of 0.8% *w*/*v*, the maximum yield of protein was obtained [30]. However, literature data [53,76] demonstrates that organic nitrogen as well as urea, nitrates, ammonium salts, and ammonia are appropriate sources of nitrogen for synthesis of proteins. It is worth emphasizing that Zheng et al. [40] found the best N:C ratios of 1:6 and 1:8 to produce SCP from *Candida* and *Rhodotorula* species using salad oil as a growing medium. For protein production, K_2_HPO_4_ and NaH_2_PO_4_ were the best phosphorus sources. The maximum protein production was reached by microorganisms cultured in the medium supplemented with NaH_2_PO_4_ 0.016% (*w*/*v*). Higher concentration of phosphorus sources decreased protein production in all experiments [30].

The wild-type yeast species can grow either in liquid medium or on the solid-state fermentation cultures. Then, yeast may be cultured in submerged fermentation, semisolid or solid-state fermentation (SSF) [12,13,73,77,78]. The choice of fermentation method depends on the choice of the culture medium. However, liquid oil substrates for oleaginous yeast are dispersed easier with moderate agitation than solid fat materials, e.g., tallow which require considerable agitation for dispersal in the culture medium [79]. Composition of culture medium has considerable effects on the rate of yeast cell growth [80]. Thus, the yield of yeast biomass and protein productivity are greatly depending on composition of growth substrate, but also culture conditions such as temperature of incubation, pH, or the moisture content of solid cultures, dissolved oxygen, and aeration content [81,82]. Therefore, the optimalization of fermentation process should be performed. There are several optimalization methods. Daskalaki et al. [83] used adaptive laboratory evolution (ALE) strategies to derive new highly productive strains. After 77 generations, the researchers obtained the strain that was able to accumulate 30% more lipids than the starting *Y. lipolytica* strain. Park et al. [84] optimized the secretory expression and purification of sweet brazzein proteins in the yeast *Kluyveromyces lactis*. The authors found that the production efficiency was highest after adding 0.1% yeast extract, 2% glycerol, and a C:N ratio of 4:1 under the conditions of 23 °C at pH 6.5. One of the more effective strategies is to use statistical experimental design techniques for selecting significant variables for delivering improved level of yield biomass [18,85]. Hezarjaribi et al. [13] used statistical techniques, e.g., the full-factorial-methodology for manipulation and optimalization of medium constituents to increase SCP production by *S. cerevisiae*. Yunus et al. [72] proved that optimalization culture conditions and medium constitution using analysis of variance test (ANOVA) significantly improved nutritional contents in yeast biomass. Furthermore, Jach et al. [81], using the statistical optimalization methods, demonstrated that small manipulation of the fermentation parameters can exert the same significant effects on protein production by *Y. lipolytica* strains without culture medium composition changes. However, Lopes et al. [18] used the statistical experimental design based on the Taguchi method, evaluating simultaneously the effect of the initial medium pH, manipulation of medium composition, and concentration of specific constituents on lipase production by *Y. lipolytica.* The most significant parameter was pH; albeit, the interaction between the medium composition and concertation of the specific constituents had the strongest effect on the production.

The medium should be sterilized to prevent contamination, especially when biomass is prepared for human nutrition. The components of the waste culture medium can be heated or sterilized by filtration. Furthermore, fermentation equipment should be sterilized as well [73]. All steps of the fermentation process should be maintained in hygienic conditions. However, yeast can grow at acidic pH (e.g., 5.0) which may contribute to the retention of possible bacterial contamination.

About 70–80% of total cell nitrogen is derived from amino acids while the remaining quantity of nitrogen comes from nucleic acids, especially RNA. The nucleic acid concentration in the yeast protein biomass is higher than in other traditional protein sources and is a characteristic feature of all fast-cultured microorganisms [7]. The biomass of most microorganisms contains 4–20% of nucleic acid [86]. However, most yeast contain low quantity of nucleic acids between 5% and 8% (lower than bacteria that contain 8–15%), which is beneficial, e.g., biomass of *Candida tropicalis* cultivated on soy molasses contained 5.28% of RNA [47]; *Candida langeronii* grown on bagasse hemicelluloses hydrolysate contained 5.8% [42]; biomass of *S. cerevisiae* as spent brewery yeast contained 8.1% of RNA [66]; and biomass of *Y. lipolytica* cultured in biofuel waste contained from 6.4% of nucleic acids in an initial lag phase, across 5.8% in the end of log phase to 0.4% at the end of stationary phase [12]. It is widely known that at the stationary phase, microbial cells have a low content of nucleic acids, which is partially caused by reduction of RNA amount [87]. Following Kurbanoglu [88], high RNA levels when consumed by humans can be toxic, but they are usually harmless for animals. In addition to providing the necessary nutrients, food should be of appropriate health quality. Therefore, single cell protein produced for human consumption should be nucleic acid-free as alkali purines (adenine and guanine) are metabolized into uric acid, which is harmful. Yeast protein should possess all desirable functional properties before its incorporation into feed and food. For improvement of yeast SCP digestibility and bioavailability, the cell wall of yeast should be destroyed by drying at high temperatures, or using mechanical forces such as crushing, crumbling, grinding, pressure homogenization, or ultra-sonification [89,90,91,92,93,94]. However, drying is one of the best methods of preserving and extending the shelf life of food [95]. In the drying method, the moisture concentration of dried yeast biomass may be lowered below 6% [12]. The moisture level below 8% in the powder is regarded as a safe for long shelf life, up to 2 years. Drying is performed before or after grinding operation and plays an important role in achieving proper texture and stability of yeast biomass as food ingredients [96]. Moreover, the dried yeast biomass obtained by drying at high temperatures (165–175 °C), which kills live yeast cells and cause cell wall disintegration, releases contents of yeast cells. The drying significantly improves quality, digestibility, and bioavailability of yeast biomass [36].

Moreover, there is a postulate that obtaining protein biomass could be improved by developing genetic engineering procedures for mass production [36]. However, it may be difficult due to the strict regulations on the use and culture of genetically modified organisms (GMO), including the cultivation of genetically modified soybeans in the European Union [71]. Therefore, the use of the wild-type yeast biomass could become helpful. Noteworthy, there is the need for large-scale production of yeast at competitive price to constitute a feasible replacement for fishmeal and soybean protein in nutrition including aquaculture [97].

## 3. Yeast Species as Protein Biomass Producers

### 3.1. Saccharomyces cerevisiae

Well-known conventional budding yeast *S. cerevisiae* (commercially known as baker’s/bakery or brewer’s/brewery yeast) is the most used source for protein production utilizing inexpensive or cost-free wastes and raw materials, especially agro-industrial and forestry substrates, as shown in Table 1. For centuries, the yeasts were the base of food processing such as baking bread or production of wine and beer, as well as a direct food source. Baking bread and alcohol fermentation are two primary processes employing the yeast, with an estimated global market value up to 9.2 billion Euro and the yearly growth forecast of about 8% [98,99]. *S. cerevisiae* is utilized to ferment the carbohydrates such as barley, corn, rice, wheat, and potatoes to produce alcoholic beverages, and in the baking industry, to expand or raise dough [100]. Other substrates include leaf juice, lignocellulosic agricultural wastes, soybean hull, starch and sugar processing wastes, fruit and vegetable wastes (fiber rich), poultry waste, spent grains, prawn-shell waste, protein rich sources, wastewaters (protein rich), waste capsicum powder, slaughterhouse, soybean meal, and combined agricultural wastes can be used for protein production by *S. cerevisiae* [101,102]. However, this yeast is not capable of fatty substrate utilization, but a transfer of the gene encoding acyl-Coenzyme A (acyl-CoA) oxidases from oleaginous yeast *Y. lipolytica* to *S. cerevisiae* enabled *S. cerevisiae* to grow on fatty acid-rich feedstock [103].

The yeast can grow at a wide range of pH (2.5–8.5) and temperatures (from 2 up to 45 °C). The tolerance of the yeast to its substrate (osmotolerance), fermentation product (ethanol tolerance), and temperature (thermotolerance) has significant potential to be used in industrial scale fermentation [104]. *S. cerevisiae* is acidophilic microorganism and grows better under acidic conditions with an optimum pH between 4 and 6 and ambient temperatures around 28–33 °C.

Commercial *S. cerevisiae* is inactive yeast, meaning dead yeast cells with no leavening power. Continuously growing fermentation industries deliver the yeast biomass as a by-product [98]. In order to improve the bioavailability of the yeast biomass, the yeast culture should undergo various treatments, such as autolysis or hydrolysis. The first mentioned method allows *S. cerevisiae* to enter the death phase when autolysis by endogenous enzymes occurs naturally. In autolysis, as yeast self-digestion, the intracellular enzymes break down glycogen, nucleic acids, proteins, and other cell components. The autolytic process needs careful application and control of heat-killed yeast cells to avoid inactivating those enzymes. The process is usually conducted under moderate agitation and temperatures between 30 and 60 °C for 12–24 h in submerged fermentation. However, there are some inconveniences in the autolytic process, such as low extraction yield, difficulty in solid–liquid separation because of high concentration of residues in autolysate, poor taste characteristics as a flavor enhancer, and risk of microbial contamination [99]. Despite these disadvantages, Podpora et al. [105] recommended the use of yeast autolysates as the best form for functional food and dietary supplement application. The authors showed that the autolysates from post-fermentation brewer’s yeast are rich in amino acids and peptides, and possess a high antioxidant potential. The most efficient method of solubilizing the yeast is hydrolysis. It is carried out by hydrochloric acid or proteolytic enzymes. In spite of a high capital production yield, acid hydrolysis is less recommended to the manufacturers due to the relatively high cost, high salt concentration, and high likelihood of carcinogenic ingredients such as 1-chloropropane and 2-chloropropane [99,106].

*Saccharomyces cerevisiae* is the most widely used yeast species in the industry. It is also a model organism used in much biotechnological research. These yeasts are used extensively in the food and feed production and pharmaceutical industries. *S. cerevisiae* has a “Generally Recognized as Safe” (GRAS) status for food products. It is a cheap and highly available source of protein and amino acids as nutritional yeast biomass or an extract as nitrogen source. Therefore, *S. cerevisiae* is an economically attractive solution for scientific studies performed for industrial purposes, with major benefits for both parties [78]. The yeast protein biomass is an attractive food and feed source replacing meat meal, fishmeal, and soybean meal for all live organisms, including fish and shrimps [107,108,109].

The yeast biomass or extract are an excellent source of B vitamins which are particularly recommended for people with increased vitamin B requirements such as adolescents, convalescents, and individuals with high physical activity [110,111,112]. The yeast extract also contains cell wall polysaccharides and carbohydrates. The high levels of carbohydrate concentration between 31% and 51% of the dry biomass were detected [66,67]. The total lipid content is low (4–7% of the dry yeast biomass) and is represented mainly by the saturated fatty acids (28–43.5% of total fatty acids) as palmitic acid (18–34%) and stearic acid (4.6–9.5%), and mono-unsaturated fatty acids (MUFAs, 62% of total fatty acids) with domination of palmitoleic acid (2.9–32%). The polyunsaturated fatty acids (PUFAs) are in low concentration 5.0–9.7% of total fatty acids, predominantly as linoleic acid (4.3%) [66,71]. The content of ash obtained from *S. cerevisiae* biomass is 5–10% [66] depending on the substrate used. The yeast biomass also contains trace minerals, including calcium, cooper, iron, phosphorus, potassium, magnesium, manganese, selenium, sodium, and zinc, and a biologically active form of chromium known as glucose tolerance factor (GTF) [66,71,99]. Biologically active GTF is trivalent form, which potentiates insulin activity, measured in vitro [113]. However, a systematic review of a randomized controlled trials of GTF supplementation as a factor in improving glycemia among patients with diabetes showed inconclusive results [11,114]. The yeast protein biomass is easily enriched with selenium (Se). *S. cerevisiae* cultivated in the presence of the inorganic selenium sources (e.g., sodium selenite) incorporates Se, primary as an organic well bioavailable form selenomethionine or selenocysteine. Inorganic Se forms in the selenized yeast biomass do not exceed 1% [115]. In humans, absorption of organic Se forms from the Se-enriched biomass is approximately 1.5–2 times higher than that from the inorganic forms [116,117]. The toxicity of organic Se forms in selenized yeast is significantly lower than that of inorganic selenite or selenate [115]. Therefore, it is recommended to not exceed the tolerable upper intake level of 300 μg/day established by European Food Safety Authority (EFSA) or 400 μg/day by the US Food and Nutrition Board. Moreover, it is worth emphasizing that the yeast biomass contains very low amounts of sodium (Na). Onofre et al. [66] determined that concentration of total Na is equal to 0.009% in spent brewery yeast biomass delivered from brewery industry.

The brewer’s yeast extract produced by enzymatic treatment is used to obtain several food processing factors such as flavoring enhancers such as monosodium glutamic acid (MSG) and nucleotides, e.g., 5′-guanosine monophosphate (5′-GMP) and 5′-inosine monophosphate (5′-IMP). Those substances, or the yeast extract, are used in meat products, sauces and gravies, soups, chips and crackers, stews, and canned food [11,99,106]. Moreover, β-glucan as a cell wall polysaccharide obtained from the yeast can be used in food products as an emulsifying stabilizer, or oil-binding, water-holding, and thickening agent [118]. β-glucan from baker’s and brewer’s yeast shows prebiotic activity; thus, it is used as nondigestible dietary ingredient with a beneficial effect on the host by selectively stimulating the growth of and/or activating the metabolism of health-promoting microorganisms in the gut [11,99]. β-glucan also may induce an immunomodulatory activity in two ways: (1) It enhances immune reactions (i.e., it exerts a prophylactic effect against common cold infection), and (2) it may reduce inflammation [11]. It is recommended to take β-glucan up to 375 mg/day as food supplements and up to 600 mg/day as nutritional food [119].

Since the yeast biomass is known as a rich natural source of protein, including enzymes, peptides, all amino acids, carbohydrates, B vitamins, and several trace minerals, and is naturally low quantity of lipids and sodium, it occupies an important place in pharmaceutical and food industry [11,66,71]. Autolysates and hydrolysates of brewer’s and/or baker’s yeast extract are used commonly as a nutrient source for the microorganism growth, especially fastidious ones or related product formations [99].

Moreover, in food products, the yeast protein biomass can replace the traditional allergic components such as sugar, dairy products, or gluten that are not tolerated and/or should not be consumed by some food-allergic people [120].

### 3.2. Yarrowia lipolytica

Non-pathogenic, oleaginous, strictly aerobic ascomycetous yeast *Y. lipolytica* is very well known for its ability to produce a large number of secreted metabolites, which are important for industry, when cultured in a wide range of hydrophilic and hydrophobic substrates, especially non-conventional ones such as plant or animal waste fats, petroleum and biofuel production wastes, and many other industrial wastes, as shown in Table 1 [11,12,15,16,17,18]. *Y. lipolytica* is also able to reduce cyanide from raw cassava which is a by-product of the bio-ethanol industry [25]. However, *Y. lipolytica* does not produce cellulases and hemicelluloses, and it is not able to reduce the content of fiber in agricultural residues [121]. The yeast can grow at a wide range of pH (2.5 to 8.0) and temperatures (from 2 to 32 °C) [122]. Its optimal growing conditions are mild acidic pH values (around 5.5) and ambient temperatures (28–30 °C) [123]. Furthermore, it tolerates metal ions, including heavy metals such as cadmium, nickel, and cobalt, and high salt concentration up to 12% (*v*/*v*) [122].

This yeast cultured in varied fatty substrates produces a lot of nutritional compounds such as protein (30–50% of dried biomass), peptides, and all essential amino acids in the right proportions (Table 1), or accumulates intracellular lipids (≥40% of cell dry weight) as single cell oil (SCO), including mono-unsaturated fatty acids (MUFAs) and saturated high added-value fats such as cocoa butter equivalents [11,15,17,18,19,28,69,70,124,125,126,127]. Protein-enriched biomass of *Y. lipolytica* cultivated in industrial glycerol contained low level of total substantial lipids (20%) in which more than 90% of total fatty acids are unsaturated fatty acids with domination of MUFAs (57.6%) predominantly represented by oleic acid (34%); PUFAs (34%) represented by linoleic (27%) and linolenic acid (6%); and SFAs in low amounts (8.3% of total lipids) [71]. In turn, protein biomasses of *Y. lipolytica* cultured in rye straw, rye bran, or oat bran-based media, contained lower concentration of total lipids (8.2–11.2% of dried biomass) with high spectrum of unsaturated fatty acids (65–78.9% of total lipids) with mostly oleic acid (37.7–59.2%); PUFAs was represented by linoleic and linolelaidic acids (11.9–19.5%) with lack linolenic acid; whereas SFAs had a higher content (19.8–34.9% of total lipids) [69]. It is worth emphasizing that the protein content of *Y. lipolytica* is comparable to that of *S. cerevisiae* biomass [11,71]. The content of ash obtained from *Y. lipolytica* when grown in industrial glycerol is 4.57% [71].

Efficient degradation of hydrophobic substrates such as n-alkanes, fatty acids, and oils by *Y. lipolytica* is a multistep process requiring a significant expansion of protein enzyme families containing paralogues of the genes involved in utilization of hydrophobic substrates [128]. After contact with lipid molecules, such as triacylglycerols (TAGs), occurs in the fatty substrates, the hydrophobic cell surface of *Y. lipolytica* secrets emulsifiers, biosurfactants, and lipases that hydrolyze TAGs into fatty acids and glycerol [129]. Among the 20 genes of lipases/esterases, *LIP2*, *LIP7*, and *LIP8* are specific for fatty acid chains of length C18, C6, and C10, respectively. *Y. lipolytica* also has 6 genes *POX* coding for the acyl-CoA oxidases. Among them, *POX2* and *POX3* code for long- and short-chain-specific enzymes, respectively. Furthermore, among the 12 genes coding for cytochrome P450 (CYP52 family), some are specific for alkanes or fatty acid hydroxylation [128,129]. Moreover, during *Y. lipolytica* growth on hydrophobic substrates, extensive peroxisome proliferation occurs. Six peroxisomal sub-forms (P1-P6) were identified, of which peroxisomes P1 and P2 underwent a fusion process followed by the successive filling in of protein matrix [128].

It is observed that the lipase content lowers in the stationary growth phase [79]. However, if an oily substrate is added, e.g., waste cooking oil as lipase inductor to the culture medium, it significantly increases extracellular lipase production by *Y. lipolytica* compared to fat-free cultures [18,130]. Thus, this yeast, cultured in hydrophobic media containing fatty acids and glycerol, is capable of intracellular lipid accumulation as storage lipid bodies, which allow yeast growth to be maintained in the absence of an extracellular carbon source [83]. When extracellular carbon source is available, at first, the yeast cultured in fatty wastes accumulate lipids as storage bodies into their cells. However, when the extracellular carbon source is depleted, the intracellular yeast lipases hydrolase lipid bodies, shifting the metabolism to the production of protein. Hence, the protein or cellular polysaccharides biosynthesis in cells of *Y. lipolytica* is in competition to the lipid storage accumulation [15,83]. It is observed that fat-free biomass production with increasing protein concentration occurs during significant degradation of storage lipid bodies and depends on nitrogen and magnesium availability in the cultured medium [83,124]. It is assumed that at higher aeration and agitation conditions, short-chain acyl-CoA oxidases exhibit increased activity. The carbon flow consequently is directed towards the synthesis of acetyl-CoA and, hence, towards fat-free biomass formation instead of storage lipophilic biomass [79]. Then, manipulation of culture conditions and fatty medium compounds, e.g., nitrogen and magnesium addition and exhaustion of the external carbon source, simultaneously contributes to obtaining the appropriate protein-enriched biomass. *Y. lipolytica* yeast can be a very good source of protein due to its specific amino acid composition (high level of essential amino acids). The amino acid profile of *Y. lipolytica* yeast is characterized by a high lysine content. Therefore, skillful design of the culture medium may result in obtaining a high yield of protein-enriched yeast biomass.

Protein biomass of this yeast is also a good source of B-complex vitamins, including vitamin B12. In similar way to animal cells, *Y. lipolytica* is able to assimilate vitamin B12 into its cells, even growing in biofuel waste [131,132,133]. The biomass contained vitamin E as well [123,131]. Furthermore, *Y. lipolytica* is a rich source of highly digestible ether extract (over 57%) when it is cultivated in industrial glycerol [71]. According to Yan et al. [134] the small amounts of the components present in crude glycerol are not particularly harmful and some (glycerides, salts, and proteins) are possibly used as combined carbon and nitrogen sources for the growth of microorganisms. *Y. lipolytica,* in similar way to *S. cerevisiae*, is able to assimilate minerals from the environment and incorporate them in its cell structures. The *Y. lipolytica* biomass contains trace minerals such as calcium, chromium, cooper, iron, iodine, magnesium, manganese, molybdenum, phosphorus, potassium, selenium, sulfur, and zinc [71,123,131,135]. The amount of sodium is low between 1.0% and 1.9% of dried biomass [131,135]. However, it is much higher than it is in *S. cerevisiae* protein biomass. Moreover, *Y. lipolytica* also contains β-glucan as a natural cell wall component [136]. Currently, *Y. lipolytica* is also used as a “workhorse” of biotechnological production: fatty acids [15,137,138]; enzymes [134]; secreted organic acids [139,140]; heterologous proteins [141]; and sweeteners [142]. Moreover, *Y. lipolytica* is an adequate model for dimorphism studies in yeast and is suited to genetic transformation, distinguishing easily its dimorphic forms, in contrast to *S. cerevisiae* which does not produce true filaments and presents pseudo-hyphae, cultured under nitrogen-limited conditions. Yeast dimorphism is likely associated with a defense mechanism to adverse environmental conditions such as nutritional changes and temperature. Additionally, *Y. lipolytica* has a reliable and versatile system for the expression of heterologous proteins for academic purposes as well as for possible commercial applications [128,138,141,143].

In nature, *Y. lipolytica* strains as living cells are often isolated from dietary products such as cheese (i.e., blue cheeses), milk, yoghurt, kefir, butter, cream, and margarine, and meat products such as various kinds of fermented sausages, e.g., salami, Spanish fermented sausages, including chorizo, and other products such as mayonnaise, seafood, juice, wine, cider, fruit, fruit concentrates, vegetables, and raw plant materials [128,141,144]. Some studies suggest that *Y. lipolytica* can be considered as normal human microbiota living as a saprophyte in the adult respiratory tract. It was also indicated in the digestive system, breast tissue, and some cutaneous nodules [145]. Moreover, the yeast strains have also been isolated from different environments such as fat-rich substrates (e.g., sewage, oily wastes), polluted soil at a car wash, seawater, and hypersaline locations [125,129,146,147].

*Y. lipolytica* is safe for humans and animals. The live yeast cells rarely cause an opportunistic infection, exclusively in individuals with compromised immunity and those with catheters [141,148]. Hence, the yeast is considered as non-pathogenic, and several production processes based on it were classified as GRAS by Food and Drug Administration (FDA) [141]. Since 2010, dried and heat-killed biomass of *Y. lipolytica* cultured in biofuel waste has been allowed to be used as a feed additive [11]. There are many studies conducted on animals such as calves [149], piglets [131,150,151], turkeys [152,153,154], and some fish, e.g., Atlantic salmon [155,156], and Pacific red snapper [157], which are enumerating nutritional benefits of the protein biomass. The quantities of essential amino acids meet the FAO requirement for fodder yeast [158]. Lastly, in 2019, EFSA has authorized the biomass as a novel food in dietary supplements for the general population above 3 years of age as safe and nutritionally advantageous. The maximum proposed daily use levels are 3 g/day for children from 3 years, up to 10 years of age, and 6 g/day thereafter. It should also be emphasized that in 2020, *Y. lipolytica* yeast biomass enriched with selenium was included in the list of authorized novel foods (food supplements) under Commission Regulation (EU) 2020/1999 (2002/46/WE) [159]. The Panel of EFSA concludes that *Y. lipolytica* biomass is safe under the proposed conditions of use. In conclusion, *Y. lipolytica* is a promising microorganism for the production of SCO and SCP. These yeasts can use various inexpensive substrates that are part of the culture medium to produce metabolites that are of great industrial importance. In addition, the use of *Y. lipolytica* as an expression or genetically modified organism makes a more and more popular system that finds interest in many research centers around the world. Such capabilities are conducive to the continuous development of biotechnology and the emergence of new challenges for the industrial economy.

### 3.3. Candida spp.

*Candida* species can use variety of substrates, have a short generation time, and their biomass is on the one hand abundant in protein [160], vitamins, and minerals but on the other hand, it contains low amounts of nucleic acids (5.8%) [42,47]. *Candida* biomass often contains a low concentration of crude oil, which makes it an appropriate low oil protein supplement for animal feed [40]. Using of *Candida* strains as SCP producers also results in a reduction of polluting parameters present in agro-industrial wastes. There are appropriate requirements for COD (chemical oxygen demand) and BOD (biological oxygen demand) that have to be met when discharging a given waste material. Thus, using *Candida* species in the production of SCP both provides valuable amino acids and enhances purification of wastes. Since *Candida* strains may absorb phenolic compounds, *Candida* biomass grown on agro-industrial wastes abundant with phenolic compounds may have an antibacterial, antifungal, antiviral, and antioxidant potential [45].

The particularly known SCP producer from *Candida* genus is *C. utilis* (also known as *Torula utilis*, *Pichia jadinii*, *Saccharomyces jadinii*, *Cyberlindnera jadinii*). This *Candida* species has been given the GRAS status and it fulfills criteria of the fodder yeast [160]. It has been used in animal feed and as a flavoring/seasoning agent in vegetarian food. Biomass of *C. utilis* is abundant in glutamic acid, which is why this species was used to replace monosodium glutamate serving as a flavor enhancer. *C. utilis* biomass is rich in essential amino acids (particularly in lysine), vitamins B (riboflavin, folic acid, nicotinic acid), ergosterol (precursor of vitamin D), and metal ions (*C. utilis* can bind them from the culture medium) [55]. This is why *Torula* yeast was used in soups and sausages in Germany during the World War I [30].

Apart from *C. utilis*, several other *Candida* species, such as *C. arborea*, *C. diddensiae*, *C. ingens*, *C. intermedia*, *C. langeronii*, *C. lipolytica*, *C. novellas*, *C. parapsilosis*, *C. pararugosa*, and *C. tropicalis*, have been already used or could be potentially used as SCP producers. These microorganisms can convert many cheap raw materials that are assimilable carbon sources into yeast biomass containing a high amount of protein. Amongst them, olive mill wastewater [45], salad oil wastewater [49], waste capsicum powder [50], potato blancher [161], potato wastewater [55], mango waste [5], poultry litter [53], sugarcane bagasse [46], soy molasses [47], rice straw hydrolysate [38], cheese whey [41], vinasse [44], prawn-shell waste [3], black liquor [162], and others have been applied. Multiple studies have confirmed that these wastes contain adequate nutrients (sources of carbon, nitrogen, phosphorus, and potassium) to cultivate *Candida* species. *Candida* strains interact differently with a given substrate (or their mixture). As a consequence, the assimilation rates of various substances as well as biomass yield are not constant for all species and applied wastes. Furthermore, total lipid and protein contents in dry biomass of *Candida* depend on composition of growth medium, including nitrogen compounds [5,50]. Some waste products serving as a medium (such as molasses) require supplementation with nitrogen or phosphorus sources [163]. It seems that the addition of ammonium sulfate to the growth medium for *Candida* is a better option than addition of ammonium nitrate [53,67]. The most preferable C:N ratio value for a high protein content is from 6:1 to 10:1. Higher values may lead to intracellular accumulation of lipids or carbohydrates which is not desired [162]. Improvement of biomass quantity and/or quality may be obtained by addition of yeast extract [5,47,53] or calcium ions [164] to growth medium from agro-industrial wastes. Several authors have noted that the addition of glycerol (e.g., 5%) to the medium containing wastes may increase a biomass yield [55] and as a consequence, a total protein yield. However, it should be mentioned that elevated concentrations of glycerol may have a negative impact on protein biosynthesis. Lower values of protein percentage in the whole biomass were noted in *C. utilis* culture when glycerol was added to potato wastewater that served as a growth medium [55]. The use of waste potato wastewater in biotechnological processes is very economical. It allows the costs of biosynthesis of many metabolic products to be reduces and reduces the amount of industrial waste, which has a positive effect on the natural environment. Natural sources of oil, coal, and gas are slowly being depleted. The need to search for alternative energy sources has contributed to the global development of the biotechnology industry. The availability and low price of glycerin from biodiesel production affects the frequent use of waste in microbiological processes. This allows the costs of biosynthesis of industrially useful compounds (e.g., organic acids) to be reduced and solves the problem with the disposal of waste from the biofuel industry. Waste glycerol can also be managed via biological methods. It is commonly used as an animal feed additive to improve its structure. Most probably, glycerol is also used for the biosynthesis of cell components other than protein, e.g., intracellular fat with nutritionally profitable fatty acids, or polysaccharides [165]. On the other side, higher concentrations of glycerol (10–25%) can inhibit the growth of *Candida* strains [55].

Other factors significantly affecting the fermentation process initiated by *Candida* strains, accumulation of protein, activation of yeast enzymes, and breakdown of ingredients in a culture medium are as follows: fermentation technique, shaking speed, moisture-to-substrate ratio, inoculum size (>7% may lead to rapid depletion in nutrients, and as a consequence to a low rate of cell survival, whereas lower concentrations may not be able to initiate the process), initial pH of inoculum (pH 3–6.2), temperature of incubation (25–35 °C), percentage of a given waste substrate in the growth medium, inoculation time (24–72 h, or even 4 days) [45,53,72,160,166,167]. Optimal values depend on a given *Candida* strain and applied medium. Usually, the protein content in yeast biomass ranges between 45% and 55%. However, depending on cultivation conditions (i.e., applied strain, medium, temperature, time) different protein levels can be obtained e.g., biomass of *Candida* cultivated in the presence of salad oil wastewater contained 26% of crude protein (*C. utilis*, 25 °C, 24 h) [49], in olive mill wastewater 35.9% (*C. pararugosa*, 30 °C, 4 days) [45], bagasse hemicelluloses hydrolysate 48.2% (*C. langeronii*, 42 °C, 30 h) [42], waste capsicum powder 48.2% (*C. utilis*, 30 °C, 48 h) [50], potato wastewater 49% (*C. utilis*, 28 °C, 48 h) [55], vinasse 55% (*C. parapsilosis*, 28 °C, 48 h) [44], glutamate fermentation wastewater 55% (*C. halophila + Rhodotorula glutinis*, 28 °C, 24 h) [40], mango waste 56.4% (*C. utilis*, 30 °C, 72 h) [5], pineapple cannery 55.3% (*C. utilis*, 30 °C, 16 h) [57], soy molasses 56.4% (*C. tropicalis*, 30 °C, 30 h) [53], rice straw 58.5% (*C. arborea*, 28–30 °C, 48 h) [38], sugarcane bagasse hemicellulosic hydrolysate 60.0% (*C. tropicalis*, 30 °C, 96 h) [48], and prawn-shell waste 60.6–70.4% (*Candida* sp., 28 °C, 7 days) [3].

Apart from protein digestibility, amino acid composition and its bioavailability belong to the most important factors regarded in food quality [168]. Most of nonruminant animals need to intake arginine, histidine, isoleucine, lysine, methionine, phenylalanine, threonine, tryptophan, and valine [44]. Protein produced by *Candida* strains contain these essential amino acids with a well-balanced composition [50]; e.g., biomass of *C. utilis* grown on waste capsicum powder is rich in lysine and threonine [50], whereas biomass of this microorganism cultivated in mango waste supplemented with external nitrogen sources contains amounts of isoleucine, leucine, lysine, phenylalanine, threonine, and tryptophan that are higher than the values recommended by FAO [5]. The biomass of the *C. parapsilosis* strain, when vinasse was used as the growth medium, contained all essential amino acids except for leucine and tyrosine, with a high content of lysine and proline [44]. Somda et al. [5] indicated that supplementing the production medium with yeast extract and ammonium sulphate may be particularly beneficial for lysine, isoleucine, phenylalanine, methionine, threonine, and valine production, which was confirmation of the results obtained by other authors [169]. Due to high lysine and threonine contents, *C. langeronii* biomass produced in bagasse seems to be a good component for feed, particularly for the ones based on cereals [42].

In SCP production, a low amount of lipids in yeast biomass is preferable. Usually, yeast contains 2–6% of lipids [45]. Biomass of *C. pararugosa* grown in the presence of olive mill wastewater has 2.8% of lipids [45], *C. utilis* cultivated in salad oil wastewater—9%, *C. utilis* grown on mango waste—4.6–13.2% [5], *C. utilis* cultivated on glutamate fermentation wastewater 0.4% [40]. The content of ash obtained from *C. utilis* cultivated on mango waste was 6.3–9.6% [5], whereas from *C. ingens* cultivated in piggery wastes 21%.

In order to increase the biomass yield and/or facilitate a given *Candida* species to grow in conditions not the most optimal (e.g., a high temperature, lower pH, inadequate carbon source), mixed cultures are a better choice. Ca. 20% increase in biomass yield and more efficient COD removal can be achieved in mixed cultures as compared to monocultures. Furthermore, mixed cultures have more advantageous amino acid compositions. Mutualism-like or commensalism-like interactions between microorganisms within mixed cultures are observed; e.g., lactose negative *C. krusei* is co-cultured with lactose-positive *K. marxianus* on cheese whey [55], or *C. utilis* is co-cultured with *Brevibacterium lactofermentus*/*Torulopsis cremoris* [170,171]. Mixed cultures of yeasts (e.g., *C. utilis* and *Endomycopsis fibuligera*, or *E. magnusi*) are used in SCP production in order to effectively and quickly convert carbohydrates from starchy materials into yeast protein. *C. utilis* does not degrade starch and it has to be co-cultured with amylolytic microorganisms [172].

### 3.4. Other Species of Yeasts

Amongst other yeasts that have a potential for the production of SCP are pentose-assimilating yeasts, quite easily prone to genetic manipulations, i.e., *Debaryomyces hansenii*, *Kluyveromyces marxianus*, and *Pichia stipitis* [173,174,175,176]. Both *D. hansenii* and *K. marxianus* have the QPS (Qualified Presumption of Safety) status [177]. Duarte et al. [59] found out that *D. hansenii*, *K. marxianus*, and *P. stipitis* are able to, in brewery’s spent grains (BGS), hydrolyzate medium (supplemented with several vitamins and microelements) with the stationary phase established after 24 h. BSG, which is usually used as animal feed or simply discarded, contains a range of nutritional ingredients that can be used for microbial growth (i.e., fiber, protein, vitamins). However, except for silicon, most of the mineral compounds found in BSG are at a very low amount (<0.5%), whereas many vitamins are partially destroyed during the hydrolysis. Both biomass productivity, yield, and ability to metabolize given compounds differed between the tested strains of *D. hansenii*, *K. marxianus*, and *P. stipitis* (*Scheffersomyces stipitis*). Glucose, xylose, arabinose, furfural acid, and formic acid were assimilated by all of them, though at various rates and quantities. It should be pointed out that only *D. hansenii* made use of all monosaccharides from the BSG medium. The highest biomass productivity and yield was also observed for *D. hansenii* [59].

*D. hansenii* is known to have minimum supplement requirements to produce a high biomass yield. It also tolerates relatively high concentrations of inhibitors. In fact, in experiments by Duarte et al. [59] the tested strain of this yeast was able to grow in BSG hydrolyzate supplemented only with KH_2_PO_4_, which from the perspective of the large-scale production is a great advantage. After a 24-h growth in the optimized BCG medium, *D. hansenii* biomass contained 31.8% of the total protein which was comparable to the values reported for other yeast strains used as source for SCP [46]. *D. hansenii* biomass had favorable fatty acid and amino acid profiles with a high unsaturated/saturated fatty acid ratio and high amounts of both essential amino acids (they meet requirements of the FAO food protein standard) and non-essential amino acids (glutamic and aspartic acid), except for methionine and cysteine which are usually marginalized in yeast proteins [42,178]. Furthermore, the obtained protein yield per sugars consumed as well as carbohydrates, RNA, and ash contents were typical for microorganisms used for similar biotechnological purposes [42,179,180].

*K. marxianus* is another yeast species attractive for industrial production due to its ability to grow on various carbon sources. It is categorized as GRAS, which is crucial for potential food and feed applications [181]. Aggelopoulos et al. [61,182] demonstrated its considerable growth on food industry wastes, i.e., a mixture of orange pulp, potato pulp molasses, and whey. The authors found out that higher amounts of orange pulp can elevate the production of *K. marxianus* biomass when cultivated in such a mixture of waste products. The obtained cell mass yield was 0.87 g/g of substrate. Orange pulp contains several vitamins B (thiamine, pyridoxine, calcium pantothenate, nicotinic acids) which are involved in cell metabolism and growth [183]. Similarly, addition of BSG to the mixture of orange pulp, potato pulp molasses, and whey promoted growth of *K. marxianus*. According to Aggelopoulos et al. [61], the ability of *K. marxianus* to grow on a mixture of various agro-industrial wastes may be used in production of the protein/lipid-enriched livestock feeds, and as a consequence, it can improve production of milk and meat. As reported, the fermented product contained 33.7% of proteins, which was over 50% higher than the protein value before addition of the yeasts. Furthermore, the fermented product contained 25.5% fat, whereas the fat content in the mixed substrates was only 3.9%. Furthermore, the fermented product contained higher amounts of magnesium [61]. *K. marxianus* was also successfully cultivated in cheese whey [41]. According to the literature data [166,184], the best parameters of growing conditions in relation to the highest yield and productivity for *K. marxianus* cultivated on cheese whey and whey permeate are: pH 4.4 and 5.8, and temperature of 31 and 30 °C, respectively. Yadav et al. [185] noted that a higher temperature and low pH may require higher energy input and may negatively influence the yeast growth. Though *K. marxianus* biomass does not contain lysine and sulfur-containing amino acids, employing a mixed culture with *C. krusei* may improve its amino acid profile. *C. krusei* contains both lysine- and sulfur-containing amino acids [55]. On the other hand, intermediate metabolites produced by *K. marxianus* may serve as carbon sources for *C. krusei* [41]. In experiments by Yadav et al. [41], the biomass obtained by co-cultivation of *K. marxianus* and *C. krusei* on cheese whey contained 43.4% of protein, 33.6% of carbohydrates, 8.4% of minerals, 6.4% of lipid, and 4.6% of crude fiber.

In the experiments by Arous et al. [45], *Schwanniomyces etchellsii* (known also as *Debaryomyces etchellsii*, *Pichia etchellsii*, *Torulaspora etchellsii*) was able to use olive mill wastewater for biomass production. However, though olive wastewater contains a high amount of nutrients and assimilable carbon sources, it is poor in nitrogen [186,187]. Since nitrogen is crucial for yeasts’ growth, it is necessary to add organic and/or inorganic nitrogen sources to olive wastewater. Using olive wastewater as an only source of nitrogen results in a low production of microbial biomass. As was noted by Arous et al. [45], a type of nitrogen source used to enrich olive wastewater significantly influences production of *S. etchellsii* biomass. The highest amounts of biomass can be obtained when ammonium chloride or ammonium sulfate is added. Other factors affecting the fermentation process are: inoculum size (5%), initial pH of inoculum (pH 5.5), temperature of incubation (30 °C), and percentage of olive wastewater in the growth medium (75%). When optimized growth conditions are provided, *S. etchellsii* biomass contains up to 39.4% of crude protein, low amounts of lipids (5%), and favorable composition of monounsaturated and polysaturated fatty acids, which could be suitable for nutritional purposes [45]. It was showed that *S. etchellsii* has a high capacity to uptake phenolic compounds (38.4 mg/g), so the yeasts biomass may have several biological activities, including antioxidant, antibacterial activities [188].

According to the literature data [189], *Aureobasidium pullulans*, a polymorphic, yeast-like fungus, could be an attractive option in SCP production thanks to its large cell size. It was shown that *A. pullulans* is able to produce significantly more cell mass than *C. utilis* when cultivated in straw hydrolysate. *A. pullulans* assimilates xylose and arabinose and it is not highly sensitive to pH changes, which is an additional advantage of this microorganism. Han et al. [190] demonstrated that the biomass of *A. pullulans* cultivated on straw hydrolysate contained a considerable amount of crude protein (42.6%) and a low level of nucleic acid (6.4%). The above-mentioned values and the amino acid profile of *A. pullulans* biomass were similar to the ones obtained for *C. utilis*.

Another interesting candidate for SCP production is *Blastobotrys adeninivorans*. It is a yeast that can utilize a variety of nitrogen-containing carbon substrates to growth. Additionally, it belongs to thermo- and osmotolerant microorganisms [191]. Lapeña et al. [35] found out that cultivation of *B. adeninivorans* on spruce hydrolysate and chicken by-products for 24 h results in a high protein content (about 50%) and low lipid (1.2%) and nucleic acids (2.8%) contents in the yeast biomass. Biomass of *B. adeninivorans*, similarly to the ones of other yeasts, is a poor source of sulfur-containing amino acids (methionine and cysteine), but it is relatively abundant in threonine and lysine. Lapeña et al. [35] presume that *B. adeninivorans* could be used as a SCP source in production of feed for animals and fish. *B. adeninivorans* biomass could provide proteins, minerals, and vitamins, but it also contains beta-glucans and alpha-mannans that have immunostimulatory properties [192].

Other yeast, such as *Hanseniaspora uvarum* and *Zygosaccharomyces rouxii*, could also be used in production of SCP from agro-industrial wastes (e.g., when cultivated on spoiled date fruit) [60].

## 4. Nutritional Benefits of Yeast Protein

Protein is considered a key component of the diet in assessing the needs of the human body. Proteins are the basic structural and functional components of every cell. They are essential for the development and metabolic processes. The importance of protein as a nutrient is to supply the body with nitrogen and certain types of amino acids. Naturally, the source of proteins and amino acids are raw materials or food products. Yeast protein biomass could contain higher amounts of protein than those obtained from the traditional protein sources such as plants or animals, as shown in Table 2. The protein amount in yeast biomass is usually similar or higher compared to protein of meat and soybean and is higher than milk protein. Moreover, the protein efficiency ratio standardized for casein (PER) of SCP yeast species compares favorably with the PER of the traditional sources of protein [65,71].

The amino acids content in protein biomass among yeast species is comparable with a high content of lysine, the most limiting amino acid in wheat, e.g., cereals [193,194]. In the yeast protein amino acids profile, the contents of isoleucine, leucine, lysine, phenylalanine, threonine and valine are higher than these required by Food and Agricultural Organization (FAO)/World Health Organization (WHO) for the human diet (Table 3). The preferable protein content is above 40% [195]. One hundred grams of yeast biomass contains a mean of 47 g of protein which corresponds exactly to almost 100% of recommended daily intake for adults (50 g) [196]. Yeast protein also contains all essential amino acids in the required quantity according to FAO recommendation [197], making yeast SCP a complete protein. It is worth emphasizing that protein deficiency may be caused not only when there is a state of relative or absolute deficiency of body proteins but also when one or more of the essential amino acids are missing [198]. Therefore, yeast biomass provides a good quality protein with healthy balance of amino acids including complete exogenous ones, being adequate for human and animal consumption.

The nutritional microbial protein value is determined by composition of amino acids, mainly 8 exogenous essential amino acids (isoleucine, leucine, lysine, phenylalanine, methionine and threonine, tryptophan, and valine) that are not synthesized by humans. Thus, these amino acids have to be supplied through the diet [7]. Containing a complete set of essential amino acids, yeast SCP is a high-quality protein source with high bioavailability for humans and animals as shown in Table 3 [11,12,14,56,71,193,199,200].

Moreover, yeast protein biomass is also a good source of macroelements such as calcium, phosphorus, and zinc, and micronutrients such as selenium and chromium. Those elements are in bioavailable organic forms found in the biomass [11,71,201]. Literature data have also indicated that yeast biomass contains a higher amount of nitrogen and ashes than fungi, algae, and/or bacteria [46]. The ash content from yeast biomass is usually between 5–10% [89], and it depends on the composition of applied medium. Furthermore, yeast protein biomass contained good quantities of B-complex vitamins, including vitamin B12 [11,81,110,111,133]. Biotin, folic acid, pyridoxine, riboflavin, thiamine, and cyanocobalamin, which are present in yeast protein biomass act important catabolic functions as coenzymes involving in carbohydrate, lipids, and protein metabolism. The adequate concentration of the vitamins is essential for optimal neurological and physiological functions of human body. It is worth noting that insufficient quantities of B vitamins are often found in diets of people living in developed countries [202]. Some yeast also produced other vitamins e.g., marine yeast *Cryptococcus aureus* G7a produced vitamin C (2.2 mg/100 g of dry weight) [58], *Y. lipolytica* biomass contained vitamin E (6.7 mg/kg of dry weight) [123,131], and *C. utilis* biomass contained ergosterol (precursor of vitamin D) [55]. Interestingly, it is observed that some yeast species, i.e., *Blastobotrys adeninivorans*, *Cryptococcus laurentii*, *Debaryomyces hansenii*, *Kluyveromyces marxianus* and *Wickerhamomyces anomalus*, possess the prion transmission reduction potential. The reuse of animal-derived wastes poses a threat for the transmission of infectious prions. Prions as misfolded proteins are responsible for neurodegenerative changes in humans and animals with a high mortality rate and their transmission may occur in breeding farms. It was detected that these five yeast species reduced Scrapie seeding activity by approximately 1 log_10_ or 90% [203].

Thus, yeast protein biomass, with its high protein content and wide spectrum of amino acids, shows very attractive nutritional benefits as a nutrient supplement for humans [11,36]. Nutritional yeast biomass is recommended particularly as food supplementation for vegan and vegetarian diets, for people who avoid eating meat, and teenagers during maturation. Commercial yeast preparations suitable for humans and animals are shown in Table S1 (Supplementary Material). The biomass can also be used by people who build muscle mass, convalescents, and those who try to prevent the risk of B vitamin deficiency [11,132,133]. Yeast protein biomass also has a higher protein-to-carbohydrates ratio than traditional forages, resulting in a better feed for livestock [30,203]. Examples of inactive yeast products for animals commercially available are presented in Table S2 (Supplementary Material). Moreover, it is worth emphasizing that yeast protein biomass, having good balance of amino acids and being rich in B vitamins, is more suitable for the feeding of poultry than plant-based feed [36]. Economic analysts’ forecasts of the technology for producing alternative protein sources (including SCP) show that in the future (by 2035) they may account for up to 11% of the global protein market [204]. In addition, the involvement of food producers, consumer behavior resulting from increased interest in health, and political aspects may contribute to the accelerated development of appropriate technology as well as production platforms that will enable the production of alternative food on a much larger scale.

## 5. Safety of Yeast Protein Used as Food

Dried and heat-killed yeast protein biomass must be safe for both human and animal nutrition in accordance with the current food and nutrition safety directives. Some studies showed that the biomasses of *S. cerevisiae* and *Y. lipolytica*, even when gown in fatty wastes, e.g., raw glycerol, did not contain excessive amounts of toxic elements such as cadmium, mercury and arsenic. The concentration of heavy metals was low and did not exceed the EU threshold values [71]. In addition, yeast protein can be Halal- and Kosher-certified by recognized organizations.

Moreover, the suitability of SCP as food must be considered individually because, like any food, it might cause allergic reactions in individuals [30,73] who are sensitive to yeast proteins. Although serious allergies are reported very rarely, subjects who are exposed to yeast inhalation are particularly at risk [115].

The consumption of yeast protein with high contents of nucleic acids could cause serious problem such as gout and kidney stone for individuals who have disfunction in purine metabolism [89,90]. Then, before consumption, yeast SCP biomass needs a reduced number of nucleic acids to 1% so it can considered as having the acceptable level of nucleic acids for using as feed or food [89]. SCP with high nucleic acid concentration is only allowed to feed animals with short life spans [91]. The reduction or removal of nucleic acid content in SCP is achieved by using various methods, such as: chemical treatment with NaOH, thermal shock, activation of endogenous nucleases (ribonucleases) in the final stage of the stationary phase by extending the yeast cultivation time or heat treatment (60–90 °C), addition of ribonucleases to the cultivation process, or it can be used as immobilized enzymes [7,12,92,93]. Moreover, for reduction of nucleic acid, Yadav et al. [94] treated the yeast biomass with two-step method with a novel combination of chemicals (N-lauroylsarcosine and NH_4_OH), which reduced the nucleic acid content to below 2%.

It is worth mentioning that *S. cerevisiae* is able to absorb and degrade mycotoxins [205,206]. Absorption of mycotoxins might occur when the yeast is cultured in fruit or vegetable wastes contaminated with molds. The brewer’s yeast biomass commonly used in food supplements are widely contaminated with ochratoxin A. Although, the level of the contamination is not assessed as high, it may bring an additional load of ochratoxin A to the total intake of mycotoxins from food. Some toxic and carcinogenic substances can arise when most microorganisms undergo sporadic mutations. Such actions can occur during unskillful processing and creation of the final product. Which can be dangerous to both humans and livestock [109]. Therefore, this should not be ignored in the case of the sensitive population such as pregnant women, children, elderly people, and immunocompromised patients [112].

Additionally, gluten-free consumers should know the difference between nutritional yeast biomass and brewer’s yeast nutritional supplements. Allred et al. [207] determined that the nutritional yeast products such as protein biomass and yeast extract contained gluten at a level below the 20 mg/kg threshold defined by the Codex Alimentarius, the Codex, or the Gluten-Free Certification Organization (GFCO) thresholds. Therefore, it is recognized that yeast protein biomass and yeast extract are gluten-free. In turn, the brewer’s yeast powder contains high levels of residual gluten derived from grain malt (e.g., wheat or barley) used to produce beer. Although, yeast organisms actively degrade any gluten in the product, there is wheat and barley gluten in spent brewer’s yeast obtained from brewing industry. Then, patients with celiac disease, who have gluten sensitivity and try to avoid gluten should be aware of the risk associated with the use of brewer’s yeast supplements.

Due to their properties, microbiological protein preparations can be used in the food industry. The search for alternative food sources (SCP), safe for human consumption, the production of which will generate low costs is very important from an economic point of view. The care for the highest quality standards of food products proves that the safety of consumers is maintained. Incompetent preparation of protein preparations may result in the presence of viral markers, allergenic substances or microbiological contamination. In this context, the emerging threats may once again pose a serious challenge aimed at conducting further research. Consumers’ acceptance is also very important while pointing out that yeast biomass can be a cheap and effective source of protein to replace common dietary protein. Despite the development of a microbiological technology for the production of food with the addition of SCP, which has a beneficial effect on the taste, consumer resistance resulting from the lack of knowledge about the existing solutions and biotechnological possibilities can be met. In this aspect, properly conducted education on the benefits of product innovations with the addition of SCP is very important.

## 6. Conclusions

Nutritional yeast biomass has high protein content and a healthy balance of amino acids, including indispensable ones, and a low quantity of lipids. The yeast protein can be added as an inexpensive supplement to the regular human diet, thus helping to solve the problem of protein/food deficiency throughout the world, and as animal feed. Yeast biomass or extract can be used in a wide range of foodstuffs as: an emulsifying stabilizer, a flavoring enhancer, and a vitamin carrier. Yeast protein is produced from a wide range of agricultural, forestry, and industrial wastes, thereby contributing to elimination of pollutants and helping in waste material recycling. With the declining acreage of farmland, problems with the emission of pollutants from animal husbandry, and the increasing number of populations, the use of yeast protein biomass as food seems to become a necessity to protect both people from hunger and the Earth from destruction. Undoubtedly, the use of alternative sources of protein resulting from the use of waste products from various industrial branches can really contribute to solving significant economic and environmental problems. In addition, an appropriate increase in the awareness and acceptance of consumers about the benefits resulting from the consumption of such a protein may improve the standard of living and a number of social phenomena that allow for the satisfaction of malnutrition. It should be noted that the search for new sources of protein and technologies for their processing requires further research.

## Figures and Tables

**Table 1:** Reports of yeast protein content produced from specific waste substrates by different yeast species.

Yeast Species	Waste Substrate	Protein Content	References
*Blastobotrys adenininvorans* (syn. *Arxula adeninivorans*)	Spruce-derived sugars and protein hydrolysates from chicken by-products	50%	[35]
*Candida* sp.	n-alkanes	65%	[36]
	Prawn-shell waste	60.6–70.4%	[37]
*Candida arborea*	Rice straw hydrolysate	58.5%	[38]
*Candida guilliermondii*	Treated distillery sludge	32%	[39]
*Candida halophila + Rhodotorula glutinis*	Glutamate fermentation wastewater	55%	[40]
*Candida krusei*	Cheese whey	48%	[41]
*Candida langeronii*	Bagasse hemicelloses hydrolysate	48%	[42]
*Candida lipolytica*	Alkaline hydrolysis of olive fruits wastes	59%	[43]
*Candida parapsilosis*	Treated distillery sludge	31%	[44]
	Vinasse	55%	[44]
*Candida pararugosa*	Olive mill wastewater	35.9%	[45]
*Candida tropicalis*	Sugar cane hemicellulosic hydrolysate (bagasse)	31%	[46]
	Soy molasses	56%	[47]
	Sugarcane bagasse hemicellulosic hydrolysate	60%	[48]
*Candida utilis*	Salad oil wastewater	26%	[49]
	Waste capsicum powder	29–48%	[50]
	Fermented rice bran	33%	[51]
	Potato starch industry waste	46%	[52]
	Poultry litter, waste capsicum powder	48%	[53]
	Potato wastewater	42%	[54,55]
	Ethanol, sulfite waste liquor	50–54%	[36]
	Tubers wastes	54%	[56]
	Pineapple cannery	55%	[57]
	Mango waste	56%	[5]
*Cyberlindnera jadinii* (anamorph name *Candida utilis*)	Spruce-derived sugars and protein hydrolysates from chicken by-products	57%	[35]
*Cryptococcus aureus*	Jerusalem artichoke extract	53%	[58]
*Debaryomyces hansenii*	Brewery’ spent grains hemicellulosic hydrolysate	32%	[59]
*Hanseniaspora uvarum*	Spoiled date palm fruit	49%	[60]
*Kluyveromyces fragilis*	Cheese whey (lactose)	45–54%	[36]
*Kluyveromyces marxianus*	Food waste mixture of orange pulp, whey, brewer’s spent grain	34%	[61]
	Cheese whey	43%	[41]
	Paneer whey	48%	[62]
*Saccharomyces cerevisiae*	Treated distillery sludge	33%	[39]
	Food waste mixture of orange pulp, molasses, brewer’s spent grain	39%	[61]
	Fruit processing residues (pineapple waste)	45%	[63]
	Fruit wastes (peels/mesocarps): mango (*Mangifera indica*), prickly custard apple (*Annona muricata*), pineapple (*Ananas comosus*), papaya (*Carica papaya*), banana (*Musa accuminara* Colla), mangosteen (*Garcinia mangostana*), cashew apple (*Anacardium occidentale*), cacao (*Theobroma cacao*), jackfruit (*Artocarpus heterophyllus*), and pomegranate (*Punica granatum*)	48%	[64]
	Vegetable processing residues (potato waste:peels)	49%	[65]
	From the beer manufacturing process	49%	[66]
	Molasses	53%	[36]
	Fruit of Beles (*Opuntia Ficus-Indica* L.) peels hydrolysate	53%	[67]
	Spruce-derived sugars and protein hydrolysates from chicken by-products	54%	[35]
*Wickerhamomyces anomalus*	Spruce-derived sugars and protein hydrolysates from chicken by-products	50%	[35]
*Zygosaccharomyces rouxii*	Spoiled date palm fruit	49%	[60]
*Yarrowia lipolytica*	Crude glycerol	30%	[68]
	Rye and oat agricultural wastes	30–44.5%	[69]
	Biofuel waste	40–50%	[12]
	Pure glycerol, raw glycerol	45%	[70]
	Industrial glycerol obtained in the production of biofuel from rapeseed	46%	[71]

**Table 2:** Average amounts of protein production of several protein sources (% dry weight) [9,12].

Organisms	Average Amounts of Protein (% Dry Weight)
Bacteria	50–65%
Yeast	29–65%
Algae	40–60%
Fungi	30–45%
Meat	45%
Soybean	35%
Milk	25%

**Table 3:** Average contents of essential amino acids in protein compared to several protein sources (milligrams of amino acids per 1 g of protein).

Amino Acids	*Saccharomyces* *cerevisiae*	*Yarrowia* *lipolytica*	*Candida* *utilis*	Wheat	Egg	Cow Milk	FAO Amino Acid Requirements for Adults
	mg/g Protein (Mean)						
Arginine	46.5	48	32	48	11.5	33	-
Histidine	23.5	26	16	16	4	37	15
Isoleucine	37	44	48	33	68	40	30
Leucine	63	68	71	67	90	88	59
Lysine	65	70	51	28	63	78	45
Cysteine	9	11	24	25	24	9	
Methionine	14	12	15.5	15	32	29	
SAA	23	23	39.5	40	56	38	22
Phenylalanine	33	40	41	45	63	47	
Tryptophan	9	47	39	11	16	Nd	
Tyrosine	26	66	20	36	19,5	16	
AAA	68	153	100	92	98.5	63	38
Threonine	48	48	41	29	50	48.7	23
Valine	53	53	55	44	74	47.9	39

AAA—Aromatic amino acids: tyrosine, phenylalanine, and tryptophan; FAO—Food and Agricultural Organization; Nd—not determined; SAA—Sulfur amino acids: methionine and cysteine.

## Supplementary Materials

The following are available online at https://www.mdpi.com/article/10.3390/metabo12010063/s1, Table S1: List of selected inactive yeast products for humans comercially available; Table S2. List of selected inactive yeast products for animals comercially available.
