# Supplementary material for: Yeast Protein as an Easily Accessible Food Source
Source: Metabolites. 2022 Jan 11;12(1):63. doi: 10.3390/metabo12010063 (PMC8780597; doi:10.3390/metabo12010063)
Supplement: Supplementary file 1 [file metabolites-12-00063-s001.zip › metabolites-1525299-supplementary.pdf]

Table S1. List of selected inactive yeast products for humans comercially available.

Name, producer and form	Composition	Recommended applications
AnPro<br>Angel Yeast<br>powder	hydrolyzed protein from brewery yeast (*Saccharomyces cerevisiae*)	It can be used in all types of foods, such as milk, energy bars, cakes, protein drinks, protein bars, meal replacement powders, and dietary supplements for vegetarians, vegans, flexitarians, and athletics, physically active people, people controlling their weight, and seniors with sarcopenia (a disorder characteristic of older people who lose muscle mass and become weaker), as well as people who care about the environment and quality products in their diet and who avoid GMO products
Brewer's yeast<br>Berfer<br>powder	Brewer's yeast (*S. cerevisiae*) rich in B vitamins and minerals	It has a positive effect on condition of the skin and hair, and improves the functioning of the nervous and digestive systems. Mannan-oligosaccharides (MOS) (MOS) present in yeast support the multiplication of the beneficial intestinal microflora, thus limiting the presence of harmful microorganisms and inflammation in the gastrointestinal tract. They prevent the formation of intestinal microflora dysbiosis.
Crisp yeast<br>Look Food<br>powder	*S. cerevisiae* rich source of B vitamins, especially vitamins B1, B2, B6, B12, niacin and folic acid, and minerals - especially zinc	A natural prebiotic - it increases the amount of beneficial bacteria from the *Lactobacillus*, *Bifidobacterium* and *Bacterioides* families in the intestines, reduce the risk of diarrhea, inflammation, polyps and neoplastic changes in the digestive tract. Prebiotics increase the absorption of calcium, iron and magnesium from food, and also have a beneficial effect on the glucose and protein metabolism in the liver. By increasing their volume, they stimulate the intestines to excrete unnecessary metabolic products. Prebiotics have a positive effect on the immune system.
Nutritional yeast<br>Bulk<br>flakes	*S. cerevisiae* rich in thiamine, niacin, folic acid and phosphorus.	The nutritional yeast is suitable for all kinds of cooking and baking. They can be sprinkled on salads or pasta, added to soups, and even used in something as delicious as vegan mac n 'cheese! Yeast flakes are an extremely versatile, convenient and tasty way to enrich your dishes with protein, fiber, vitamins and minerals. Nutritional yeast gives food a cheesy, nutty flavor and is extremely versatile for vegan and vegetarian.
Nutritional Yeast Flakes<br>Now Foods<br>flakes	Brewer's yeast (*S. cerevisiae*) with additional B vitamins	Naturally rich in vitamins and minerals.
Levadura de Cervesa<br>El Naturalista<br>powder	brewer's yeast (*S. cerevisiae*) rich in phosphorus, potassium, iron and calcium and B vitamins (B1, B2, B3, B5, B6, B8, B9 and B12).	It is recommended to eat to taste, it can be added to meals or juices.<br>Brewer's yeast is rich in biotin (an important vitamin for creating hair, skin, and nails). It also contains essential amino acids essential to human life and necessary for the production of red and white blood cells. Due to the content of chromium, it improves carbohydrate metabolism and increases muscles and increases energy supply, which is why many athletes include them in their diet. It is also associated with a decrease in postprandial glycemia and a reduction in triglycerides and an increase in HDL.
Nutritional Bland Yeast<br>Energy<br>homogeneous flakes.	Dried brewer's yeast (S. cerevisiae) in a refined beet mix	It has a delicious, slightly salty taste, not bitter as opposed to traditional yeast. It is perfect for healthy people and convalescents, children, athletes, vegans and vegetarians. It is an exceptional source of high-quality protein and rich in all B vitamins.<br>It can be sprinkled directly on salads and hot and cold dishes. It is used for making vegan cheeses, for casseroles, as a filling ingredient. It can also be combined with spices or herbs to make spices. Improve the flavors of dishes, sauces, pasta, pizza. It can be used to enrich soups and creams. In vegetable smoothies and beverages, such as pouring morning cereal.
Yeast extract<br>Different companies e.g.	Yeast extract (*S.cerevisiae*)	B vitamin source for vegetarian.

Marmite or Meridian bread spread		
Saccharomyces Max Solherbs tablets	Dried brewer's yeast (S. cerevisiae) rich in fiber and protein, B vitamins, and minerals: potassium, phosphorus, calcium, magnesium, zinc, iron	It support acne therapy. It is also used in the treatment of seborrhea, hyperkeratosis of the epidermis or stretch marks. It exhibits antioxidant properties. Inactivated yeast relieves abdominal pain especially in people suffering from irritable bowel syndrome. Chromium contained in yeast reduces the level of total cholesterol in the blood, and chromium, together with zinc, supports the regulation of blood sugar levels. Potassium in yeast has a beneficial effect on blood pressure.<br>For those who want to lead a healthy lifestyle as well as for people with special nutritional needs, i.e. vegans, the elderly, convalescents, athletes, people striving to maintain a healthy body weight as well as people with skin problems.
Brewers yeast star StarLife tablets	brewer's yeast, vitamin B3 (niacin), vitamin B1 (thiamine hydrochloride), vitamin B2 (riboflavin)	Protein and B vitamins source
Brewer's Yeast Lifeplan Tablets.	brewer's yeast *(S. cerevisiae* Vitamin B1, vitamin B2, vitamin B3	Over-excitability, cracking corners of the mouth, skin problems, fatigue, lack of appetite, anaemia.
Brewer's Yeast Swanson Tablets	brewer's yeast *(S. cerevisiae)*, vitamin B2 (riboflavin), vitamin B12	It is a powerful energy booster that supports healthy sugar metabolism.
Drovit 400 mg Drofarm Jaworzno Tablets	Brewer's yeast *(S. cerevisiae)*	Weight-loss diet and in fatigue and weakness as well as in problems with skin, nails and hair.<br>The formulation supplements the daily intake of B vitamins and magnesium.
Dromin Intenso Apipol Farma Tablets	*S. cerevisiae,* zinc, vitamin B1, vitamin B2 3,2 mg, vitamin B3 32 mg, vitamin B5 12 mg, vitamin B6	Problems with seborrhoeic or mixed complexion, deterioration of skin condition and weakening of hair and nails.<br>The formulation supplements of everyday diet with nutrients that help maintain proper condition of the skin, hair and nails. The manufacturer recommends it especially to mature women.
Dromin Apipol Farma Tablets	*S. cerevisiae*, flower pollen extract, vitamin B1, vitamin B2, phosphor, magnesium, potassium 60 mg, calcium, zinc	Active growth of adolescents, weakness resulting from an unbalanced diet and fatigue, conditions associated with skin problems.<br>Thanks to the content of free amino acids, vitamins and microelements, the formulation facilitates normal course of metabolic processes in the body. Due to the content of flower pollen extract, the manufacturer warns against using the product in individuals allergic to bee products.
Humavit N Varia tablets	*S. cerevisiae*, vitamin B6, vitamin B3	Excessive hair loss, fatigue, weakness and in brittle nails.<br>The supplement prevents the formation of perleche and seborrhoea, and enhances physical and mental health, relieving fatigue and weakness.
Humavit V Varia	*S. cerevisiae*, vitamin A, vitamin C, vitamin E	Replenishment of the diet with B vitamins (thiamine, riboflavin, niacin), minerals (calcium, iron, magnesium, zinc, copper) and vitamins A, C and E.

tablets		The composition of the product ensures its favourable effect on the skin, hair and nails. Furthermore, the formulation can prevent the development of perleche and seborrhoea, improve metabolic processes and support the body's immunity. It is particularly recommended to tobacco smokers, consumers of high-fat meals, and the elderly as well as during a convalescence period.
Humavit Z<br>Varia<br>tablets	Brewer's yeast *(S. cerevisiae)*, horsetail (extract), nettle (extract)	Fragile and brittle nails, hair loss.<br>The product exerts a favourable effect on the condition of the skin and mucous membranes and facilitates excretion of detrimental metabolic waste products from the body.<br>The formulation replenishes everyday diet with B vitamins and mineral salts (derived from brewer's yeast) and silicon compounds (derived from horsetail and nettle).
Humavit Z Plus<br>Varia<br>tablets	*S. cerevisiae*, horsetail (extract), nettle (extract), D-biotin, whey protein complex	The formulation replenishes the diet with the ingredients that exert a favourable effect on the condition of hair and nails. It is used as an adjunctive treatment in acne vulgaris.<br>The manufacturer warns that the product is intended for adults only.
Lewipollen<br>Centuria<br>tablets	*S. cerevisiae,* vitamin B1, vitamin B2, vitamin B3, flower bee pollen, chromium,	General fatigue, lack of appetite, skin problems (acne, eczema, furunculosis, cracking corners of the mouth, complexion problems in adolescence), problems with hair (excessive hair loss) or nails.<br>The product is abundant with easily absorbable natural B vitamins, derived from brewer's yeast, that facilitate metabolic processes of the body (metabolism of carbohydrates, fats and proteins) and support its immunity.<br>The manufacturer does not recommend taking the formulation to individuals allergic to bee products
Lewitan AO<br>Centuria<br>Tablets	*S. cerevisiae,* vitamin B1, vitamin B2, vitamin B3, biotin, phosphorus, potassium, powdered angelica root	The formulation exerts a favourable effect on the nervous system, skin, hair and nails, and it can facilitate digestion process. In addition, it also alleviates migraine. It is also recommended in states of general fatigue, exhaustion, and as a stimulant of the body's defence mechanisms.
Lewitan MOL<br>Centuria<br>Tablets	*S. cerevisiae,* Vitamin B1, vitamin B2, vitamin B3, chromium, powdered lemon balm leaf,	The formulation is recommended especially in states of general fatigue, exhaustion, as a stimulant of the body's defence mechanisms, and in problems with the skin, hair loss and brittle nails. Furthermore, the product exerts a sedative effect and regulates functioning of the digestive system.
Lewitan MP<br>Centuria<br>Tablets	*S. cerevisiae,* vitamin B1, vitamin B2, vitamin B3, chromium, powdered mint leaf,	General fatigue, exhaustion, weakening of the body's defence mechanisms, problems with the skin, hair loss and brittle nails.<br>The product exerts a favourable effect on the gastrointestinal tract, increasing the secretion of digestive juices and eliminating flatulence.
Lewitan Z<br>Centuria<br>Tablets	*S. cerevisiae,* vitamin B1, vitamin B2, vitamin B3, chromium	Complexion problems, nagging perleche in their mouth corners, acne vulgaris,<br>The product is also recommended in order to improve metabolism, as well as during active growth of adolescents, in times of weakness resulting from an unbalanced diet and fatigue, and in hair loss caused by the weakening of hair follicles.
Vegavit Witamina B12<br>Lanes<br>Tablets	brewer's yeast *(S. cerevisiae)*, vitamin B12, brewer's yeast	Vegetarian or vegan diet<br>The product replenishes the diet with B12 vitamin and other B vitamins found in brewer's yeast.
Yeast With Pansy<br>Colfarm	yeast extract *(S. cerevisiae)*, viola tricolor herb extract, vitamin E,	The product exerts a favourable effect on the appearance and condition of the skin. Yeast extract helps maintain smooth and radiant complexion, especially in adolescence. The formulation is intended especially for individuals

Tablets	organic zinc	who care about the appearance of their skin, hair and nails.
Selenium&Zinc<br>Power Health Products Ltd<br>Tablets	brewer's yeast *(S. cerevisiae)*, selenium's yeast 100 µg of selenium, zinc oxide 2 mg of zinc	All the active substances of the formulation are of natural origin and are easily absorbed by the body. Selenium and zinc support immunity, combat free radicals, prevent premature aging, protect the heart and the circulatory system.
Selenium yeast supplemented with flower pollen<br>Gal<br>capsules	*S. cerevisiae*, flower pollen, zinc, pantothenic acid, vitamin B1, vitamin B2, folic acid, selenium	Fatigue and exhaustion , problems with the skin, hair loss and brittle nails.
Inter Yeast - Vital<br>Inter Yeast<br>tablets	*S. cerevisiae* rich in B vitamin and minerals	Blood lipid metabolism, skin and hair.
Olimp Labs Immubiotic<br>Olimp Labs<br>capsules	zinc-enriched yeast, selenium-enriched yeast (*S. cerevisiae),* lyophilized of probiotic bacteria strains: *Lactobacillus acidophilus Rosell-52*, *Lactobacillus rhamnosus Rosell-11*, *Bifidobacterium longum Rosell-175*	Weakened immunity, risk of increased susceptibility to viruses and bacteria (autumn-winter season), periods during and after antibiotic therapy.<br>The product contains 3 selected bacterial strains that support the body's natural defences during viral and bacterial infections, increased physical effort or stress, and maintain a balanced intestinal microflora during and after antibiotic therapy. In addition, the dietary supplement contains zinc and selenium in the organic form, derived from yeast biomass enriched with minerals.
Red yeast rice<br>Now Foods<br>capsules	fermented red rise by *Monascus purpureus* containing monacolin K	It helps to maintain a normal level of cholesterol in the blood. Monacolin K reduces the production of cholesterol by the liver.

Table S2. List of selected inactive yeast products for animals comercially available.

**Product name, producer, form**	**Composition**	**Recommended applications**
Animal feed Candida 45%<br>Henan JDZ Bio-Engineering<br>powder	Torula yeast (*Candida utilis*)	Protein source for all animal species.
Technoyeast<br>Biochem<br>powder	hydrolyzed *Kluyveromyces fragilis*	Protein source rich in essential amino acids such as the umami-tasting glutamic acid and yeast cell wall components as mannan oligosaccharides (MOS) and β-glucans and yeast extract for weaned piglets a significant increase in average daily feed intake and weight gain.
Brewer's yeast<br>Gempol<br>powder	brewer's yeast *Saccharomyces cerevisiae* rich in B vitamins and amino acids	Protein-vitamin and therapeutic supplement to the main diet of animals, fish and birds. It helps increase weight gain, increase milk production of goats, cows, increase egg productivity of hens, ducks, geese and helps improve quality of wool.

Biergiest Proteine<br>QUIKO - Beer Yeast<br>powder	brewer's yeast *S. cerevisiae* rich in B vitamins	Protein source for all ornamental bird species.
Vitawel<br>powder	*S. cerevisiae* CBS, *Enterococcus faecium* NCIMB, pea protein extracts, plant extracts	Prevention of diarrhea in calves and support of the digestive system. It improves the digestibility of milk. It has a positive effect on the weight gain of the animal. Application to milk from birth to a minimum of three weeks of age.
Peloton<br>PMI Nutritional Additive<br>powder	*S. cerevisiae*	Maintaining rumen health and digestive efficiency.
Brewer's yeast<br>Barf<br>powder	brewer's yeast *S. cerevisiae*	A supplement for dogs and cats, enriching the diet with a number of vitamins, minerals and amino acids. Brewer's yeast is highly bioavailable, has a beneficial effect on the digestive system, and improves the condition of the skin and fur.
InterYeast<br>InterYeast<br>powder	brewer's yeast *S. cerevisiae*	Protein source for all animal species.
Yeast<br>Nuvena Adds<br>powder	*S. cerevisiae* rich in B vitamins and minerals	For the health of the entire horse body, and in particular for the functioning of the digestive system. They improve the use of feed, improve the appearance of hair and hooves, have an effect on the nervous system and help build muscle mass.
Yea-Sacc 1026<br>Dodson&Horell<br>powder	*S. cerevisiae* 1026	Supplement naturally supporting the digestive system and improves fiber digestion and increases bacterial fermentation in the cecum for horses.
Yeast feed<br>Vetos Farma<br>powder	*S. cerevisiae*	Concentrated feed additive for all types and species of farm animals, especially for young animals, pregnant females and animals undergoing veterinary treatment.
YeaFI BM<br>Leiber<br>powder	brewer's yeast *S. cerevisiae,* malt sprouts	Supporting digestive processes and cellular metabolism of all livestock: cattle, pigs, poultry and pigeons, a positive effect on fertility and improve the general condition of animals, stimulation of oestrus symptoms in sows (reduction of silent oestrus), improved feed conversion and weight gain, reduction of losses during piglet rearing and weaning.
Biomin<br>Biomin<br>powder	*S. cerevisiae*<br>An autolytic degradation of the yeast cell content provides functional components such as the ß-glucans and mannan-oligosaccharide cell wall compounds, ribonucleic acid (RNA), nucleotides, amino acids and peptides (amino acid chains) in a pre-digested form.	Feed additives routinely supplemented in cattle. These bioactive components serve as nutritional sources for beneficial anaerobic rumen microbes such as fiber- and cellulose digesting bacteria that digest the forage driving ruminant nutrition as well as stimulating lactate-utilizing bacteria thus helping avoid acidic rumen conditions such as sub-acute rumen acidosis (SARA).

YeaSel<br>Angel<br>powder	*S. cerevisiae* rich in selenium produced by submerged fermentation of *S. cerevisiae* in selenium-enriched media	Improve animal reproductive performance; relieve stress; reduce somatic cell counts in milk; improve carcass quality. For: piglets, finishing pigs, sows and boars, broilers, layers.
Zorien SeY<br>Novus<br>powder	*S. cerevisiae* enriched with selenium in an organic form	It is highly bioavailable, and can help maximize animal performance by promoting the body's natural defense system for all animal species.
Alkosel Selenium Yeast<br>Lallemand Animal Nutrition<br>powder	selenium enriched *S. cerevisiae*	Protein and L(+)-selenomethionine source for use in pig, poultry, and cattle (dairy and beef) feeds.
Yarrowia Canifelox Gastro<br>Skotan<br>powder	*Yarrowia lipolytica* A-101, sea algae, milk thistle, probiotic bacteria (*Lactobacillus acidophilus, Enterococcus faecium, Pediococcus acidolactici, Lactobacillus casei*).	A formula supporting proper digestion in dogs, recommended for adult dogs with gastrointestinal disorders. *Y. lipolytica* yeast in combination with 4 strains of probiotic bacteria is an excellent synbiotic that promotes the multiplication of useful intestinal microflora. Thanks to this, the preparation improves digestion and absorption of nutrients, as well as reduces the amount of diarrhea and has a prophylactic effect, preventing their occurrence.
Equinox Respiratory<br>Skotan<br>Liquid	*Y. lipolytica* A-101 with selenium, rice bran, magnesium chelate, calcium carbonate, spirulina, hawthorn, vitamin E, manganese chelate, fruit flavor	A support in the treatment of cough and respiratory allergies for horses .
Yarrowia Equinox Classic<br>Skotan<br>powder	*Y. lipolytica* A-101, calcium carbonate,dried apples, fruit flavor.	During periods of increased load, it improves the body's efficiency and endurance and affects the rapid regeneration of muscle fibers for horses.
Yarrowia Equinox Gastro<br>Skotan<br>powder	*Y. lipolytica* A-101, calcium carbonate, ethyl esters of Omega-3 / -6 / -9 fatty acids, mint, fennel, licorice root, chamomile, anise	Supports the healing of stomach ulcers by reducing the symptoms of peptic ulcer syndrome in horses. It strengthens immunity, improves digestion, increases efficiency, strengthens the hair and hoof horn, regenerates the body's cells. It quickly and effectively compensates for mineral deficiencies, supports the vitality and condition of the body, has prebiotic properties for horses.
Yarrowia Equinox Sport<br>Skotan<br>powder	*Y. lipolytica* A-101, calcium carbonate, dried apples, dried carrots, vitamin E, vitamin C, sodium selenate.	A prebiotic effect, support metabolism, the work of the digestive tract and the immune system. They also have a positive effect on the condition of the coat and hoofs. The product is highly digestible and easily accessible to the body for horses.
Yarrowia Equinox Hooves<br>Skotan<br>powder	*Y. lipolytica* A-101, calcium carbonate, dried apple, magnesium chelate, linseed oil, dried raspberry and parsley leaves, buckwheat, zinc, copper and manganese chelate, fruit flavor, sodium selanate, biotin, purple echinacea, vitamin C;	For horses with hoof problems. It causes fast growth of the hoof horn, prevents mycosis and infections of the dart, and prevents hoof fractures.

Yarrowia Equinox Electrolite Skotan powder	*Y. lipolytica* A-101, calcium carbonate, sodium chloride, potassium chloride, magnesium chelate, glucose, vitamin E (alpha-tocopheryl acetate) and vitamin C (L-ascorbic acid)	An electrolytic preparation that replenishes the level of lost electrolytes, effectively reducing the risk of dehydration. The active ingredients of the preparation support the perfect condition of the body and quick regeneration after intense exercise.
